# Metformin is associated with survival benefit in pancreatic cancer patients with diabetes: a systematic review and meta-analysis

**DOI:** 10.18632/oncotarget.15692

**Published:** 2017-02-25

**Authors:** Ping-Ting Zhou, Bo Li, Fu-Rao Liu, Mei-Chao Zhang, Qian Wang, Yan-Yan Li, Ci Xu, Yuan-Hua Liu, Yuan Yao, Dong Li

**Affiliations:** ^1^ Department of Oncology, Shanghai Ninth People's Hospital, Shanghai Jiaotong University School of Medicine, Shanghai, China; ^2^ Department of Bone Tumor Surgery, Changzheng Hospital, Second Military Medical University, Shanghai, China; ^3^ Department of Chemotherapy, Nanjing Medical University Affiliated Cancer Hospital, Cancer Institute of Jiangsu Province, Nanjing, Jiangsu, China; ^4^ Department of Radiation Oncology, Shanghai Ninth People's Hospital, Shanghai Jiaotong University School of Medicine, Shanghai, China

**Keywords:** metformin, pancreatic cancer, diabetes, overall survival

## Abstract

**Background:**

Pancreatic cancer is a highly lethal disease with a poor prognosis while metformin has been associated with a decreased risk of pancreatic cancer. Although the benefit of metformin was observed for pancreatic cancer prevention, it is not clear whether it can also affect the survival of pancreatic cancer patients with type 2 diabetes mellitus. A systematic review and meta-analysis was conducted to assess the effect of metformin on the survival of pancreatic cancer patients with type 2 diabetes mellitus.

**Methods:**

Two independent authors searched PubMed and Web of science up to 08/07/2016. We assessed studies for eligibility, extracted data, and examined their quality, with the primary outcome as overall survival. We used published hazard ratio (HR) available or estimated based on other survival data. We pooled the data and used a random-effect model to combine direct comparisons from included articles. We also investigated treatment effects by different countries, quality and the time of metformin initiation.

**RESULTS:**

We found that there was a relative survival benefit associated with metformin treatment compared with non-metformin treatment in both overall survival (OS) ([HR] 0.84; 95% confidence interval [CI]: 0.73 – 0.96). These associations were also observed in subgroups of Asian countries and high quality articles.

**Conclusions:**

Our results support the notion that metformin maybe the best anti-diabetic medicine of choice in patients with pancreatic cancer and concurrent type 2 diabetes mellitus. The perspectives of enhancing survival of pancreatic cancer patients with diabetes mellitus by the use of metformin deserve more attention in future research and clinical practice.

## INTRODUCTION

Pancreatic cancer (PC) is the fifth leading cause of cancer-related death in developed countries [1], and it is projected that the incidence is to increase by 55% from 2010 to 2030 [2]. PC is one of the most lethal types of cancer, with a 5-year survival rate of <5% [3, 4], mainly because of the late detected stage and a shortage of well characterized biomarkers for the disease [5–7]. Smoking is the most common risk factor for PC, and other risk factors include heavy alcohol consumption, chronic pancreatitis, and dietary/endocrine factors [8]. Recently, several studies have reported that obesity and type 2 diabetes mellitus (DM) are associated with increased incidence for PC [9, 10].

Therapies for diabetes include exogenous insulin, sulfonylureas and metformin. Metformin is the most commonly used therapy for DM worldwide [11]. It works mechanistically by reducing insulin resistance and decreasing hepatic glucose production. Metformin has been associated with a decreased risk of breast cancer [11, 12], gastric cancer [13]and ovarian cancer [14]. Recent epidemiological studies also showed that metformin might decrease the risk of PC incidence [15, 16].

Although the benefit of using metformin was observed with PC prevention, it is not clear whether it can also effect on the survival of PC patients with DM. Since 2009, a growing number of articles focus on the effectiveness of metformin on survival of PC patients with DM, but the results are remarkably inconsistent. Here we conduct a systematic review and meta-analyses of the literature to better understand the effect of metformin on the survival of PC patients with DM.

## RESULTS

### Selection of articles and characteristics

**Figure 1:**
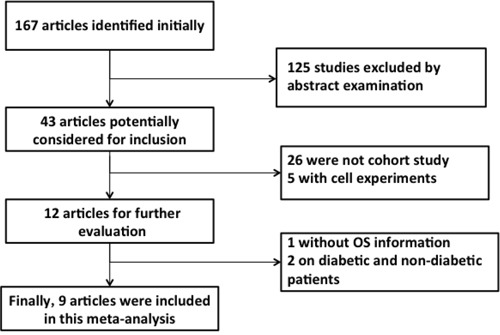
Flow diagram of study identification

The selection of relevant articles is depicted in the flow chart (Figure 1). Briefly, we first identified 43 trials, of which 31were excluded because they did not fulfill the inclusion criteria.12 trials [17–28] were potentially suitable, but 3 trials [26–28] were eventually excluded because they didn't provide complete information. A total of 9 cohort studies [17–25] were available for inclusion in the quantitative analysis of the survival benefits of metformin for PC patients concurrent with DM, with one study [25] including two comparisons. Finally, 10 comparisons were included in our meta-analysis.

The total number of individuals in the studies was 9, 265 patients with PC and DM. 4871 patients received metformin alone or in combination with other anti-diabets medicine while the rest (4394 patients) received non-metformin treatment such as thiazolidinedione, insulin, sulfonylurea, thiazolidinedione, and dipeptidylpeptidase-4 inhibitors. The characteristics of the studies are summarized in Table 1. Included studies were retrospective cohort design and clinic-based setting. According to the Newcastle-Ottawa Scale (NOS) the quality of 6 studies [17–20, 24, 25] was high.

**Table 1:** Characteristics of the studies included in the meta-analysis

Study	Year	Country	Design	NOS	Sample Size(met/non-met)	OS	Mean fllow-up
Amin et al.	2016	USA	Cohort	7	1916(1098/818)	Y	NR
Chaiteerakij et al.	2016	USA	Retrospective cohort	6	980(366/614)	Y	9.26 months
Ambe et al.	2016	USA	Cohort	7	44(19/25)	Y	19 months
Lee et al.	2015	Korea	Retrospective cohort	6	237(117/120)	Y	10.3 months
Choi et al.	2015	Korea	Cohort	4	183(56/127)	Y	10.2 months
Hwang et al.	2013	USA	Retrospective cohort	5	516(247/269)	Y	NR
Nakai et al.	2013	Japan	Retrospective cohort	3	124(8/116)	Y	9.9 months
Sadeghi et al.	2012	USA	Retrospective cohort	8	302(117/185)	Y	11.4 months
Currie et al.	2012	UK	Cohort	6	5016(2843/2173)	Y	NR

Abbreviations: met, metformin; non-met, non-metformin; NR, not reported.

These studies were conducted in America and Europe (6 studies, 94.7%) [17–19, 22, 24, 25] or in Asia including Japan and Korea (3 studies, 5.3%) [20, 21, 23], without studies from other Continents. The male proportion (54.9%) included in the studies is slightly higher than female, with a mean age of 69±8.8 years. Most studies excluded older people and individuals with clinically significant comorbid conditions. Three studies [17, 22, 25] performed analysis comparing patients who had received metformin before PC diagnosis. Meanwhile, patients in three studies [18, 20, 25] had DM at the time of PC diagnosis. The median follow-up data in the trials were mature, and median follow-up time ranged from within 9.26 months [18] to within 19 months [19].

### Overall and subgroup analysis

**Figure 2:**
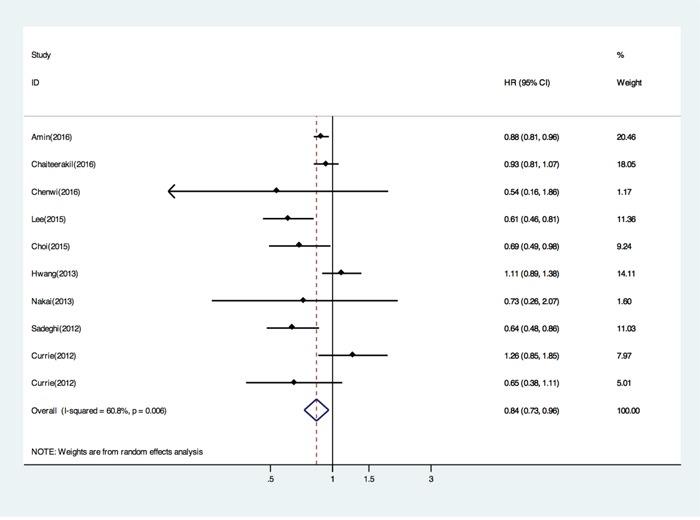
Forest plot (random effects model) of pooled Hazard ratios(HRs) of overall survival by metformin versus non-metformin treatment Each study is shown by the point estimate of the HR (the size of the square is proportional to the weight of each study) and 95% CI for the HR (extending lines). The diamond represents the summary HR and 95% CI.

The survival analysis was based on 9 trials, 9318 patients, and 657 deaths. When compared with non-metformin treated patients, the pooled HR of death was 0.84 (95% CI, 0.73 to 0.96; *P*, 0.01) for metformin users (Figure 2). This corresponds to an absolute survival benefit at 16% with metformin. We next performed subgroup analysis by countries (Asian or Western countries). In the subgroup of Asian countries, metformin was still associated with reduced death risk by fixed model (HR 0.65; 95% CI: 0.52 – 0.80; *p* 0.830 for heterogeneity; *I^2^* 0%) (Table 2). However, no significant difference was observed in the relative survival benefit with respect to the subgroup of Western countries (HR 0.90 by random model; 95% CI: 0.78 – 1.03; *p* 0.028 for heterogeneity; *I^2^* 57.6%) (Table 2). Metformin efficacy was found to be significantly superior for PC in the subgroup of 6 high quality studies (HR 0.81; 95% CI: 0.70 – 0.95; *p* 0.012 for heterogeneity; *I^2^* 63.3%) (Table 2), but in 3 low quality studies benefit was not significant (HR 0.88; 95% CI: 0.60 – 1.29; *p* 0.067 for heterogeneity; *I ^2^* 63.1%) (Table 2). We also performed subgroup analyses to examine the effects of metformin according to timing of metformin initiation. Metformin use before PC diagnose showed no benefit of survival (HR 0.89; 95% CI: 0.83 – 0.96; *p* 0.02 for heterogeneity; *I ^2^* 60.8%) (Table 2).

**Table 2:** Pooled analysis of association of metformin use and survival of PC concurrent with DM

	N	Sample size	HR	*P*_h_	*I^2^*
**Total**	10	9318	**0.84**	0.006	60.8%
			**(0.73-0.96)**		
**Country**					
Asian	3	544	**0.65(0.52-0.80)**	0.83	0.0%
Western	7	8774	0.90(0.78-1.03)	0.028	57.6%
**Quality**					
High	7	8495	**0.81(0.70-0.95)**	0.012	63.3%
Low	3	823	0.88(0.60-1.29)	0.67	63.1%
**Exposure**					
Before PC diagnose	3	4740	1.00(0.81-1.24)	0.1	56.6%
After PC diagnose	3	2359	0.76(0.52-1.11)	0.028	72.0%
**Publication bias test**					
Begg's test		*p*=1.00			
Egger's test		*p*=0.896			

*P*_h_: *P* for heterogeneity.

### Sensitivity analyses and publication bias

**Figure 3:**
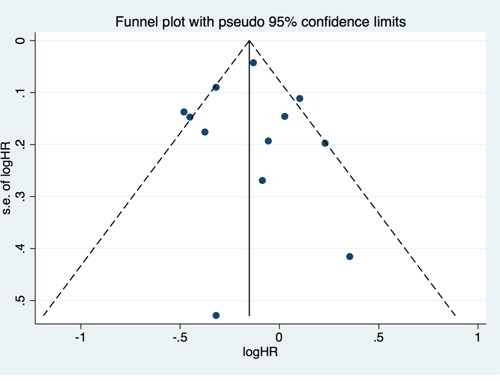
Funnel Plots to detect publication bias Each circle represents an individual article. HR indicates Hazard ratio. Circle represents identified studies.

There was substantial statistical heterogeneity between studies with an *I^2^* value of 60.8% (*I^2^* test for heterogeneity, *P* 0.006), which could be explained partly by study location (Western vs. Asian). No evident of publication bias was discovered by Begg's and Egger's tests for total mortality (Figure 3). All *p* values for a two-sided test were >0.1. In addition, visual inspection of the funnel plot did not reveal remarkable asymmetry. We also carried out sensitivity analysis and found that omission of any study at a time did not alter the pooled results even if the most influential study was omitted.

## DISCUSSION

To our knowledge, our meta-analysis is the first quantitative study to inspect the association between metformin treatment and survival of patients with PC and type 2 DM concurrently. Our study included 9318 patients from 9 independently published investigations, and the pooled results provided evidence that the use of metformin is associated with improved OS in the treatment of patients with PC and type 2 DM.

Pancreatic cancer can lead to DM by destroying islet cells or by causing peripheral insulin resistance. Islet amyloid polypeptide, the concentrations of which are elevated in PC patients with DM, appears to be responsible for the insulin resistance [29]. Strikingly, nearly 60% of PC patients with new-onset of DM, who underwent pancreatico-duodenectomy, had resolution of their DM following tumor resection [30–32]. These results suggest that new-onset DM in PC patients is likely a consequence of the cancer. On the other hand, it has been reported that about 1% of DM patients being diagnosed within 3 years were diagnosed with pancreatic cancer in a population-based cohort of 2122 people over 50 years old [33]. Long-standing DM is a risk factor for pancreatic cancer, and there is indeed an increased incidence of development of pancreatic cancer among patients with long-standing DM [32, 34].

Preclinical studies have suggested that anti-diabetic medicine may influence the risk of cancer. Metformin, the most widely used oral anti-diabetic drug worldwide, might decrease the risk and mortality of cancer in patients with DM and therefore has facilitated numerous preclinical and clinical studies to investigate its anticancer activity [35–37]. Most proposed mechanisms of metformin can be attributed to inhibiting respiratory complex I of the mitochondria, thereby reducing oxidative phosphorylation, ATP production and ROS production in cells [38–42]. This in turn triggers the activation of adenosine monophosphate–activated protein kinase (AMPK), a cellular “energy thermostat” that plays a key role in regulating energy metabolism [43]. Anticancer role of metformin was related to the activation of AMPK and consequent inhibition of the mammalian target of rapamycin (mTOR) pathways that are frequently present in malignant cells [44–48]. In addition, it has been shown that metformin can inhibit pancreatic tumor growth in obese, prediabetic mice through inhibiting transforming growth factor-beta (TGF-beta), epithelial-to-mesenchymal transition (EMT) and regulating expression of Notch in pancreatic cancer cells via targeted binding of miR-34a [49]. Although the mechanisms by which metformin influences tumor growth have recently been reviewed, it is uncertain whether the use of metformin can lead to better clinical outcomes for PC patients concurrent with DM.

Several studies have reported that patients receiving metformin show a significantly increased survival compared with breast, ovarian cancer patients with DM without metformin treatment [50–52]. Retrospective studies comparing survival outcomes between metformin and non-metformin treatment in PC patients with DM have appeared up to 2012. In our meta-analysis the included patients have concurrent pancreatic cancer and DM, avoiding diabetes itself as a confounder. We selected OS, which has been the gold standard for demonstrating benefit of clinical medicine as endpoints. We designed a comprehensive search strategy and examined relevant resources to retrieve potentially eligible trials. A total of 9318 patients with pancreatic cancer and concurrent type 2 diabetes were included in the meta-analysis. We found that metformin use is associated with a decreased mortality of PC. Compared with non-metformin treatment, metformin treatment with an OS benefit was equipped with a 14% reduction of death in the patients (HR 0.84; 95% CI: 0.73 – 0.96). This result was also supported by our subgroup analysis demonstrating significant difference in high quality studies (HR 0.81; 95% CI: 0.70 – 0.95) and Asian countries (HR 0.65; 95% CI: 0.52 – 0.80). Although our meta-analysis shows that the use of metformin is associated with an increase of pancreatic cancer survival among Asian individuals, we found no such association in Western countries. The differences observed in Asian countries against the Western countries could be attributed to differences in diet and life styles. In addition, Asian subjects, possibly attributable to having increased insulin resistance, hyperinsulinemia and central obesity, might benefit from several of the putative benefits of metformin therapy [53]. Together, these factors might lead to the different effects of metformin on PC patients in Asian and Western groups.

With respect to survival, the disregard of the initiation time of metformin use may bring about unintended biases, leading to the over estimation of the drug's effect. We perform subgroup analysis by initiation time of metformin use, of 9 eligibly articles, 5 cohorts [17, 18, 20, 22, 25] examined the impact of metformin use classification on survival. The results suggested that metformin use either before or after PC diagnose also has no significant benefit in survival (HR 1.00; 95% CI: 0.81 – 1.24; HR 0.76; 95% CI: 0.52 – 1.11). The results from these studies might be affected by inadequate statistical power, because they had only limited sample size in the subgroups and are prone to incomplete information about the initiation time of metformin use.

There are several limitations inherent to our meta-analysis of the published studies. First, according to NOS, three comparisons were assessed as low quality, thus restricting the interpretation of the results. Subgroup analysis show that metformin use lost significant benefit in 3 low quality publications. On the other hand, high quality studies confirmed the findings of metformin benefit. Second, following the criteria, we included only a limited number of trials (n=9). The low number of included articles weakens the validity of our results. Third, the reported meta-analysis was limited by substantial heterogeneity among the studies (*p* .006 for heterogeneity; *I^2^* 60.8%). Subgroup and sensitivity analyses were performed to evaluate the effect of potential effect modifiers. The high heterogeneity of results is probably because the populations used in studies were from various countries. Lastly, the results may be influenced by the different levels and durations of metformin exposure. It is extremely difficult to determine the beneficial dose and duration of metformin for improved survival because few studies have provided the related information.

Based on the meta-analysis results, our study demonstrates that metformin use in individuals with PC and concurrent type 2 DM can increase OS compared with those without the use of metformin. Our results suggest that metformin might be the anti-diabetic medicine of choice in patients with pancreatic cancer and concurrent type 2 DM.

## MATERIALS AND METHODS

### Study selection

Two researchers independently conducted a systematic literature search to identify studies that investigated treatment of PC patients with DM with metformin. All relevant articles of English language published before August 2016 were identified from PubMed and Web of Science. The search terms used were “metformin”, “diabetes”, “prognosis or survival or mortality” and “pancreatic cancer or neoplasm or carcinoma or adenocarcinoma or tumor”. We reviewed all articles and potential studies referenced for additional consideration. In addition, we searched all relevant review articles.

Studies included in this meta-analysis should meet the following criteria: 1) they contained hazard ratios (HR) and 95% CI about survival or mortality of disease; 2) they included randomized, controlled trials (RCTs); and 3) they also included observational studies. Exclusion criteria were: 1) no data about survival parameters in the abstract; and 2) *in vitro* or animal studies.

### Data extraction and quality assessment

Data extraction from text, tables, and figures of included studies was carried out independently by two investigators (Zhou PT and Li B) using standardized forms. Discrepancies were resolved by consensus in consultation with the third author (Li D). A quality assessment of the studies was performed to understand the bias in individual studies. The methodological quality of the observational studies was assessed by two authors independently (Zhou PT and Li B) using the Newcastle – Ottawa scale [54].

### Data analyses

We compared the outcomes of ratios in patients treated with metformin versus a control group (placebo or other anti-diabetics medication). For the network meta-analysis of overall survival data, we applied a random-effect to calculate the HR estimates and 95% CI as previously described [55]. Subgroup analyses were conducted for country location of the studies, article quality and history of metformin used in patients.

We evaluated the between-study heterogeneity using a Cochran Q test, with a *p*-value <0.10. We also operate heterogeneity with *I*^2^ test and we considered *I^2^*<30% as representing low statistical heterogeneity and *I^2^*> 75% as representing high statistical heterogeneity [56].

We investigated the publication bias for our meta-analysis by using a funnel plot and the asymmetry by using Begg and Egger tests [57, 58]. Finally, we performed sensitivity analysis by removing each study at a time to evaluate the stability of the results. All analyses were performed using Stata version 12.0 software (StataCorp, College Station, TX).
